# Rewarming Injury after Cold Preservation

**DOI:** 10.3390/ijms20092059

**Published:** 2019-04-26

**Authors:** Thomas Minor, Charlotte von Horn

**Affiliations:** Department for Surgical Research, University Hospital Essen, Hufelandstr. 55, D-45147 Essen, Germany; charlotte.von-horn@uk-essen.de

**Keywords:** temperature paradox, rewarming injury, organ preservation, controlled rewarming, COR

## Abstract

Organ dysfunction pertinent to tissue injury related to ischemic ex vivo preservation during transport from donor to recipient still represents a pivotal impediment in transplantation medicine. Cold storage under anoxic conditions minimizes metabolic activity, but eventually cannot prevent energetic depletion and impairment of cellular signal homeostasis. Reoxygenation of anoxically injured tissue may trigger additional damage to the graft, e.g., by abundant production of oxygen free radicals upon abrupt reactivation of a not yet equilibrated cellular metabolism. Paradoxically, this process is driven by the sudden restoration of normothermic conditions upon reperfusion and substantially less pronounced during re-oxygenation in the cold. The massive energy demand associated with normothermia is not met by the cellular systems that still suffer from hypothermic torpor and dys-equilibrated metabolites and eventually leads to mitochondrial damage, induction of apoptosis and inflammatory responses. This rewarming injury is partly alleviated by preceding supply of oxygen already in the cold but more effectively counteracted by an ensuing controlled and slow oxygenated warming up of the organ prior to implantation. A gentle restitution of metabolic turnover rates in line with the resumption of enzyme kinetics and molecular homeostasis improves post transplantation graft function and survival.

## 1. Introduction

Preservation/reperfusion injury still represents a major denominator of graft dysfunction after transplantation. Limited quality of grafts from extended criteria donors, warm ischemia upon donation after cardiac standstill and extended times of preservation are frequent exponents that adversely affect the functional resilience of the organ during the period of ex vivo storage. It seems likely, that the existing preservation technology should be further developed to comply with the future requirements in the evolving field of graft preservation and conditioning. To this prospect, a thorough understanding of pathophysiologic issues pertinent to preservation/reperfusion injury will be crucial. Recent experimental evidence is suggesting that the abrupt shift in temperature occurring during transit from cold preservation to normothermic reperfusion triggers tissue injury and consecutive graft dysfunction after transplantation. In the following, we will hence give a synoptic perspective on emerging knowledge concerning the role of this “rewarming injury” in the context of organ preservation, molecular backgrounds and first strategies to minimize its repercussions in clinical routine.

## 2. Cold Ischemic Preservation

Conventionally, means to minimize graft injury during this period between organ retrieval and transportation have been found in cooling the organ to temperatures between 0 °C and 4 °C [1]. According to van’t Hoff’s equation, there is an approximate decrease of the metabolic rate in the tissue of 50% for any decrease in temperature by 10 °C [2]. Thus, hypothermic storage, itself, will reduce cellular metabolism by little more than the factor 10. In this regard, the use of substrates that are lacking after cessation of nutritive perfusion will be economized and the shut-down of energy-requiring homeostatic processes is postponed.

However, the above mentioned metabolic retardation is a diversified effect, depending on the activation energy of individual reaction processes and whether they are enzymatic or non-enzymatic. Membrane bound chemical reactions are more affected than cytosolic ones, due to a cold induced loss of fluidity of lipid membranes that hamper conformational changes of inbound protein complexes [3].

In the long run, the residual metabolism even at 4 °C will suffer from the lack of oxygen [4], being the first missing and by far most important substrate to fuel energy production for basic cellular needs.

Depending on the disposition of the graft and the severity of the ischemic challenge, this can eventually result in tissue injury and organ dysfunction upon reperfusion after transplantation.

However, although cellular homeostasis is progressively disturbed during cold ischemic preservation, the definitive impact on functional recovery will, for the most part, only manifest during the initial phase of reperfusion [5]. With respect to the clinically used time limits of hypothermic organ preservation, ischemic cellular disarrangements only trigger the ulterior tissue damage incurring upon re-introduction of oxygen after transplantation which then impairs immediate graft recovery and also affects long term outcome after transplantation.

## 3. Reoxygenation Injury

Re-introduction of oxygen into anoxically injured tissue not only replenishes cellular energy stores and re-establishes metabolic homeostasis. At the same time, it paradoxically fuels the production of oxygen free radicals (OFR) that directly cause structural damage to tissue membranes, DNA or proteins. Additionally, oxidative stress is recognized as a trigger to apoptotic transformation and cell death [6].

This so-called oxygen paradox [6] mainly acts on three sources of OFR generation: Breakdown products like xanthine or hypoxanthine, that unnaturally accumulate during anoxic catabolism of high energy phosphates [7] can be metabolized at very high rates by xanthine oxidase (XOD), a radical generating isoform of the enzyme xanthine dehydrogenase (XDH) induced during ischemia [8]. Another reaction that generates OFR, not requiring the conversion of XDH into XOD, is a rapid re- oxidation of NADH instead of xanthine by human xanthine dehydrogenase fostered by increased levels of reduced nicotinamide adenine dinucleotide (NAD) [9]. However, the most critical source of OFR in ischemia reperfusion injury lies in disturbed respiratory chain reactions at the mitochondrial site [10].

Anoxia leads to a large depletion in ATP and a maximal reduction of the electron carrier pool. Intermediates of the citric acid cycle accumulate, and namely succinate increases as a characteristic signature of ischemia. [11]. At reperfusion, succinate will serve as electron store to be rapidly re-oxidized by reverse electron transport (RET) at complex I, as forward reactions at the respiratory chain are hampered by excessive amounts of reduced coenzyme Q [12]. This reverse action, however, is accompanied by a substantial generation of superoxide anion radicals.

Complex I, a protein complex and part of the electron transport chain at the inner mitochondrial membrane, converts into a deactivated state during ischemia [13] but gets rapidly reactivated upon reoxygenation by conformational transition and can then generate OFR through the above mentioned RET reactions. Pharmacological blocking of complex I in the deactivated state can inhibit reverse electron transfer from the reduced NADH pool downstream of complex I upon reoxygenation, although metabolic intermediates are not yet balanced and, thus, mitigate production of OFR [14].

In this regard it is tempting to assume, that hypothermic conditions during reoxygenation might also exert a protective effect by slowing down the process of re-conversion of complex I into its active form.

Interestingly, reoxygenation in the cold can reduce OFR mediated tissue injury upon later reperfusion at normothermia [15,16] and effectively mitigate reperfusion injury after transplantation [17,18,19] as has been demonstrated in different animal models of liver or kidney preservation.

Moreover, experimental data in rat livers show that the temperature of oxygenated reperfusion after cold preservation is directly correlated with the amount of cell injury [20]. While there was virtually no enzyme leakage upon reperfusion at 4 °C, the tissue injury increased 20-fold at 20 °C and to about 50-fold upon reperfusion at 37 °C. Based on these observations, the ‘*oxygen paradox*’ pairs with a ‘*temperature paradox*’ and abrupt rewarming should be considered a genuine mechanism of tissue injury during preservation/reperfusion of organ grafts.

## 4. Rewarming Injury

In an isolated permanently oxygenated perfused liver model, the mere rise in temperature from 4 °C to 37 °C entailed a significant impairment of the respiratory control ratio and a dramatic decrease in tissue ATP content [21]. As oxygen was abundantly present throughout the experiments, this effect must be attributed to the rise in temperature itself.

The above mentioned temperature paradox seems to be mitigated by slowing down the process of tissue rewarming during a period of ex vivo perfusion [22]. In a direct comparison, gradual increase of organ temperature by controlled oxygenated rewarming (COR) during machine perfusion has shown to significantly reduce tissue injury upon warm sanguineous reperfusion when compared to a similar protocol in which machine perfusion was only executed in the cold or no machine perfusion was carried out at all.

Adapting the thermal kinetics to a slow and progressive restoration of mitochondrial energy homeostasis may help avoid transient metabolic disarrangements and injury to the cell, associated with abrupt reperfusion [23].

**Figure 1:**
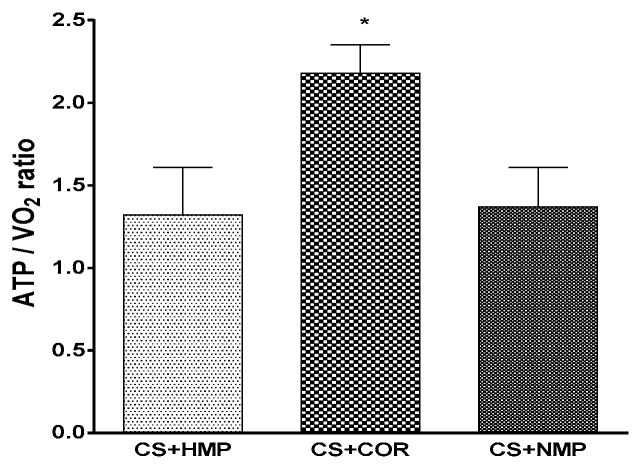
Efficiency of oxygen utilization as measured by tissue content of adenosine triphosphate (ATP) in relation to total oxygen consumption upon isolated reperfusion after 18h of cold storage and 90 of reconditioning machine perfusion; CS+HMP: oxygenated hypothermic machine perfusion; COR: controlled oxygenated rewarming prior to warm reperfusion, i.e., gradually increasing temperature over time; NMP: normothermic machine perfusion. (* *p* < 0.05 vs other groups, Student–Newman–Keuls test).

Actually, it appears that COR is, at least in part, operative to improve mitochondrial efficiency upon reperfusion as the ratio of built up ATP and the accompanying consumption of oxygen shows up to be optimized by gradual instead of abrupt rewarming (cf. Figure 1).

Hypothermia is thought to result in a progressive solidification of lipid membranes and thereby disturb conformational reactivity of membrane-bound enzyme systems [3]. Thus, it might be conjectured that the re-establishment of proper membrane fluidity may take more time than the swift increase in metabolic activity upon immediate normothermia would allow to fulfill the urgent requirements upon warm reperfusion. In consequence, a defect membrane function hence would entail cellular dysfunction at a time when rewarming, especially after ischemic preservation dramatically increases energy demand of the tissue and the immediate ‘ad hoc’ functional response of mitochondria is pivotal to prevent reperfusion injury.

Small lipid fragments, e.g., HNE (4-hydroxy-2-nonenal), that are generated via radical peroxidation of lipid membranes upon reoxygenation, were shown to induce proton leaks across the mitochondrial membrane mediated through induction of uncoupling proteins and interference with the adenine nucleotide translocase (ANT) [24]. Free iron that seems to be required for these reactions [25], frequently becomes available during longer periods in hypothermia [26].

Interestingly, a significant reduction of lipid peroxidation and accumulation of HNE upon reperfusion of kidney grafts could be obtained by the use of COR as compared with abrupt restoration of normothermia and this actually went along with a substantial normalization of the overall efficiency of oxygen utilization in the organ [27]. Although not directly shown on a subcellular level, this study suggested an increase in futile oxygen consumption by uncoupled mitochondria to be secondary to abrupt rewarming of cold tissue, which can be, at least in part, prevented by a gradual and adapted rise in temperature.

A central role in mitochondrial dysfunction upon ischemia reperfusion is played by the opening of the mitochondrial permeability transition pore (MPTP) [28]. The onset of the mitochondrial permeability transition (MPT) is fostered by mitochondrial generation of reactive oxygen species, an increase of mitochondrial free Ca2+ and the immediate restoration of cellular pH back to normal values upon reperfusion along with an increased efficiency of pH-dependent phospholipases or proteases that had been largely inactive at acidotic pH during ischemia [6,29].

The net consequences of MPT comprise mitochondrial depolarization, uncoupling of oxidative phosphorylation [30] and the release of pro-apoptotic proteins from the intermembrane space, such as cytochrome c.

Free cytosolic cytochrome c in turn triggers the activation of initiator caspase 9 and induction of apoptotic cell death via caspase 3. The timing of this process has been shown to be very consistent with the act of rewarming [31]. In parallel, nicotinamide adenine dinucleotides (NAD) that are leaking through the open pores are thus becoming available for enzymatic hydrolysis by glycohydrolases localized outside the mitochondrial matrix space [28]. Moreover, under these circumstances NAD may be washed out during reperfusion and is no longer at the disposition/available for restoration of adequate amounts of energy by respiratory chain reactions.

Most, if not all, of these events are not predominantly linked to the mere re-introduction of oxygen into cold, energy depleted tissue, but unveil only upon and due to the abrupt resumption of high metabolic activity triggered by the swift change to normothermia at the time of reperfusion.

## 5. Mitigation of Rewarming Injury

Actually, short-term hypothermic oxygenation after the preservation period reduces functional disturbances, structural injury and whole graft dysfunction upon ulterior warm reperfusion [32,33,34].

Notwithstanding that, residual injury incurring upon abrupt rewarming of the graft might only be counteracted by controlling the rise in temperature using an adapted slow rewarming perfusion protocol prior to reperfusion.

Mitigation of the temperature shock by such measures indeed prevented or alleviated generation of OFR, mitochondrial loss of NAD [35] and the activation of caspase 9 [36].

Other preservation protocols, including continuous oxygenated hypothermic machine perfusion during the whole preservation period, came up with sensibly inferior results [35], suggesting that even optimal preservation prior to reperfusion does not eliminate the susceptibility to rapid warming up from deep hypothermia. Meanwhile, several groups have confirmed positive effects of gradual warming up of organ grafts prior to warm reperfusion. In porcine liver transplantation, Matsuno et al. [37] reported serum levels of transaminases when gradual rewarming perfusion was used during preservation instead of continuously hypothermic machine perfusion. Isolated rat kidneys showed less injury to renal tubular cells if gradual warming up was implemented before warm reperfusion [38]

Encouraging results have also been observed in the clinical setting. Liver grafts that were offered by rescue allocation after being rejected for transplantation by other centers had been subjected to the COR-treatment and all of them were successfully transplanted [39]. The protocol is now under way to be further evaluated in a randomized controlled trial (CORNET-study ISRCTN94691167).

In a similar approach, de Vries and coworkers have taken human discard livers donated after cardiac arrest of the donor and put them on a machine perfusion apparatus for sequential cold and warm perfusion, using a period of controlled oxygenated rewarming between cold and warm perfusion [40]. Based on specific parameters observed during isolated perfusion, five out of seven livers were then judged of adequate viability and three-month graft survival after transplantation was 100%. Earlier experiments on controlled rewarming have been carried out using conventional cold preservation solution as a perfusate for the temperature regulated machine perfusion. Since these media are not conceived for the use at elevated temperatures, the rewarming process had been limited to mid-thermal temperatures [22] for not taking the risk of treating the graft at normothermia with inadequate media. The question, if an extension of the rewarming protocol to temperatures at normothermia might enhance the protective potential of the method has later been addressed using a serum-like perfusion solution (Aqix RS I™), the buffering capacities of which allowed for a stable pH over the whole temperature range from hypo- to normothermia [41].

In a direct comparison, using the same solution for controlled rewarming perfusion up to 20 °C or up to 35 °C, no additive protection could be evidenced for the latter. The critical range for the temperature paradox to occur thus seems to be between 4 °C and 20 °C. Hence, the adapted thermal control seems basically needed during the hypo- and mid-thermic range while above 20 °C further warming up appears to be relatively well tolerated by the graft.

Supportive data for this conclusion come from experiments in isolated rat hepatocytes. Longer periods of incubation at temperatures between 4 °C and 16 °C actually elicited a significant rewarming injury upon abrupt return of the incubation temperature to normothermia while virtually no such reaction was triggered after incubation at temperatures above 16 °C [26].

Nonetheless, the enhanced rewarming protocol might be of clinical interest for pre-transplant evaluation and viability assessment of organ grafts. The physiologic temperature conjecturally represents a more appropriate condition for assessing functional status of marginal organ grafts [41] and does probably also increase the efficiency of additional pharmacotherapy, e.g., immunomodulation, genetic therapy or measures to improve regeneration, all of which will become interesting goals in the future [42].

Another even more radical approach to avoid the rewarming injury upon transplantation has been pioneered by the Oxford group, who has established a complete preservation method by continuous up-front normothermic machine perfusion (NMP) [43,44]. The superiority of NMP over conventional cold storage has been documented in several experimental studies and recently been clinically confirmed in a large randomized controlled trial [45]. NMP maintains a fully functioning state of the organ during the whole ex vivo period and thus precludes longer periods at hypothermia as well as the associated cold induced tissue alterations.

Besides a global reduction of structural and functional impairments of graft upon reperfusion, experimental data disclosed a significant reduction of mitochondrial loss of cytochrome c along with a net reduction of apoptotic transformation [46]. This mechanistic observation is well in line with the avoidance of the above described disarrangements attributed to the rewarming injury.

Additionally, a mitigation of pro-inflammatory reactions upon reperfusion has been described after organ preservation with NMP comprising a reduction of free radical mediated lipid peroxidation, and lower levels of IL6 and danger associated molecular patterns (DAMPS) at the time of reperfusion [47].

It is of interest, that this dampening of innate immune reaction and inflammatory response after NMP occurred to about the same extent (about 50%) than it had been described after COR which was also operative in reducing TNF [22,48], HMGB1 and the extracellular matrix protein tenascin C [49] to approximately half of the values observed after static cold storage alone.

However, a direct comparison of NMP and COR is still missing and will surely come up with valuable insights into the respective efficiency to reduce preservation and/or rewarming injury to transplanted organ grafts.

Since NMP is only fully operative, when effectuated from retrieval to implantation [36,50], the method requires trafficking of sophisticated machine devices for NMP to the place of organ retrieval and the necessary surveillance during transport to the recipient further add to complicate the traditional logistics for organ retrieval and transportation.

Similar considerations apply to the recent technique of subnormothermic oxygenated machine preservation [51,52]. While also preventing notable periods of hypothermia and, thus, presumably precluding major risk of rewarming injury, continuous subnormothermic perfusion still requires attendance during transport of the organ and involves circumstantial effort.

By contrast, the controlled use of machine assisted oxygenated warming up in an ‘in house’ condition at the implantation clinic has a much smaller footprint on organ retrieval logistics and furthermore minimizes the risk of organ loss due to malfunction during transport.

## 6. Conclusions

For a better understanding of tissue injury during and after organ preservation it is important to acknowledge the difference between reoxygenation injury as known from warm ischemic pathologies in vivo and the more complex situation upon ex vivo organ preservation in transplantation medicine.

In the latter, the protective effect of reducing cell metabolism during ischemia by cooling is linked to the fact that a large temperature step is to be made at the time of reperfusion. This potentially dissociates two distinct phenomena, i.e., an oxygen paradox on one hand and a temperature paradox on the other. Thus it opens the possibility to introduce oxygen while hypothermic suppression of metabolic turnover minimizes re-oxygenation injury. However, then full metabolism only recovers if, in a second step, normothermic conditions are re-established in the tissue.

Gentle adaptation of this rewarming process to most carefully meet the respective homeostatic requirements during the thermal transition phase will be chance and challenge of the future.

## Abbreviations

ANT	Adenosine nucleotide transporter
ATP	Adenosine triphosphate
COR	Controlled oxygenated rewarming
MPT	Mitochondrial permeability transition
NAD	Nicotineamide dinucleotide
NMP	Normothermic machine perfusion
OFR	Oxygen free radicals
HNE	4-Hydroxy-2-nonenal
RET	Reverse electron transport
XDH	Xanthine dehydrogenase
XOD	Xanthine oxidase
